# NR4A1 destabilizes *TNF* mRNA in microglia and modulates stroke outcomes

**DOI:** 10.1371/journal.pbio.3002226

**Published:** 2023-07-25

**Authors:** Yao Yao

**Affiliations:** Department of Molecular Pharmacology and Physiology, Morsani College of Medicine, University of South Florida, Tampa, Florida, United States of America

## Abstract

Microglia play a dual role in stroke depending on their pro-inflammatory and anti-inflammatory polarization. This Primer explores a PLOS Biology study that identifies a new mechanism through which the transcription factor NR4A1 negatively regulates TNF expression in microglia.

## Teaser

Microglia play a dual role in stroke depending on their pro-inflammatory and anti-inflammatory polarization. A study in *PLOS Biology* identifies a new mechanism, through which the transcription factor NR4A1 negatively regulates TNF expression in microglia.

Microglia are brain-resident immune cells that represent 5% to 12% of the glial population [1]. Under physiological conditions, microglia take a ramified morphology (small cellular body and long processes) and constantly survey the brain microenvironment by extending and retracting their processes [1,2]. Upon injury or in neurological disorders, microglia quickly change to an ameboid morphology (large cellular body and short processes) and alter their transcriptional profiles [1,2]. Depending on the inflammatory mediators secreted, reactive microglia can take a pro-inflammatory or anti-inflammatory state. The former functions to kill pathogens, while the latter is involved in debris clearance and injury repair [1,3]. Imbalance between the pro-inflammatory and anti-inflammatory states causes damage and affects injury resolution [1,3]. What regulates the pro-inflammatory and anti-inflammatory polarization of microglia, however, is not fully understood.

Nuclear receptor subfamily 4 group A member 1 (NR4A1), a transcription factor and immediate early gene, is rapidly induced in macrophages and microglia. Functional studies have revealed an anti-inflammatory role for NR4A1 in macrophages in both atherosclerosis and chronic inflammation models [4–6]. Similarly, NR4A1 inhibits microglial activation and pro-inflammatory gene expression in animal models of Parkinson’s disease and multiple sclerosis [7,8]. These findings suggest that NR4A1 restricts unrestrained inflammation in microglia and macrophages. However, the underlying molecular mechanism remains largely elusive.

**Fig 1:**
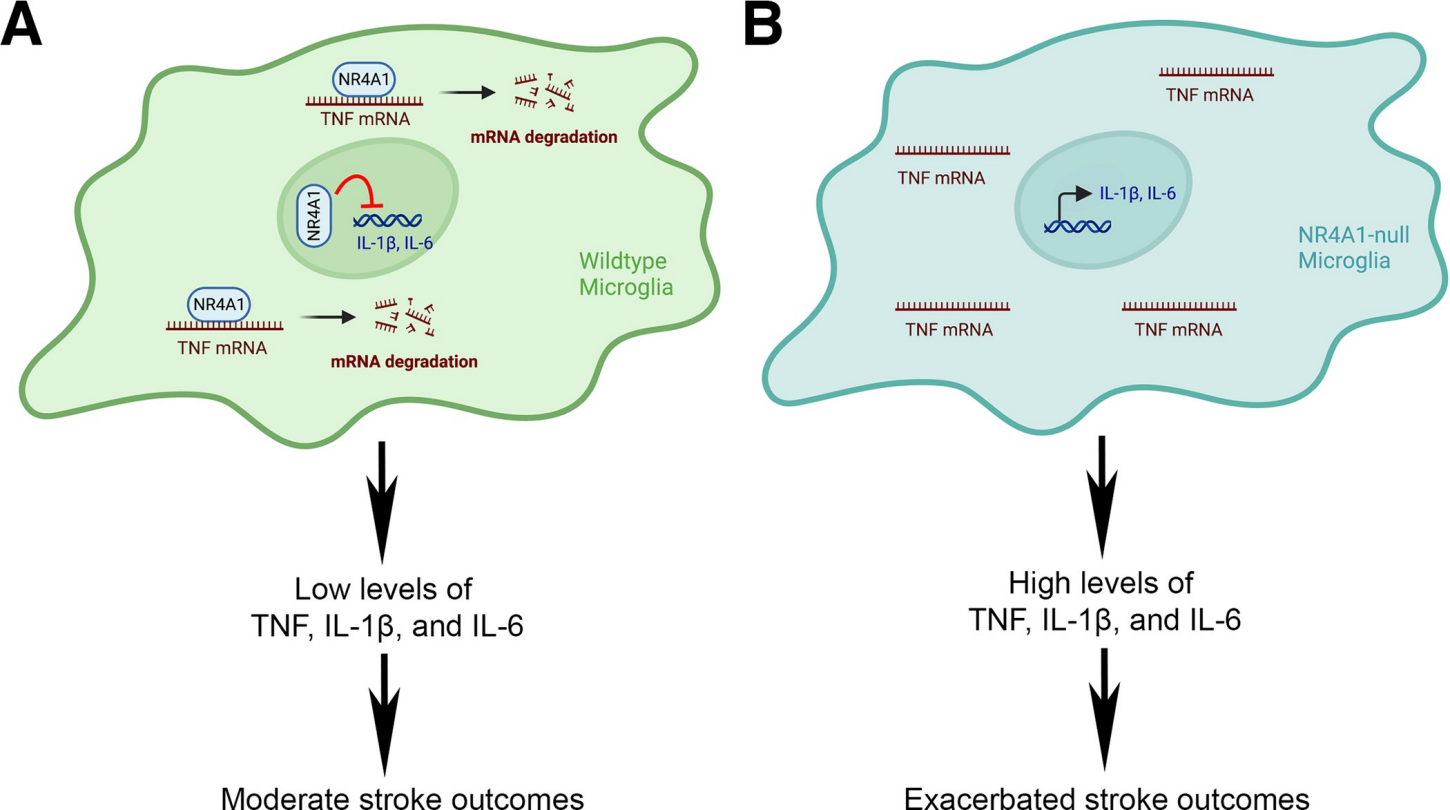
NR4A1 plays a neuroprotective role in stroke by regulating the expression of pro-inflammatory cytokines in microglia. (**A**) In the cytoplasm, NR4A1 diminishes the expression of TNF posttranscriptionally by binding to and destabilizing *TNF* mRNA. In the nucleus, NR4A1 negatively regulates the synthesis of IL-1β and IL-6 at the transcription level as a transcription factor. Thus, wild-type microglia produce relatively low levels of pro-inflammatory cytokines, leading to moderate stroke outcomes. (**B**) Loss of NR4A1 in microglia increases the production of pro-inflammatory cytokines (TNF, IL-1β, and IL-6), leading to aggravated stroke outcomes. This figure was created with BioRender.com.

In this issue of *PLOS Biology*, Liu and colleagues [9] report a novel posttranscriptional mechanism that mediates the regulatory effect of NR4A1 on the expression of pro-inflammatory cytokine-tumor necrosis factor (TNF) in microglia (Fig 1). Specifically, the authors find that cytoplasmic NR4A1 directly binds to and destabilizes *TNF* mRNA in an N6-methyladenosine (m^6^A)-dependent manner. Next, they show that NR4A1 is up-regulated in the cytoplasm of activated microglia and localizes to processing bodies (P-bodies) in vitro. Consistent with the in vitro results, NR4A1 levels are increased in microglia after ischemic stroke in both rodents and humans. Using NR4A1 global knockout mice, Liu and colleagues further demonstrate that loss of NR4A1 leads to increased TNF expression and worse outcomes in a mouse model of ischemic stroke. Since NR4A1 is also expressed in non-microglial cells, including macrophages and T cells, the authors further generate mutant mice with NR4A1 deficiency in microglia only using the microglia-specific TMEM119-CreER^T2^ line. Similar to the NR4A1 global knockout mice, loss of NR4A1 in microglia specifically results in increased TNF expression and aggravated outcomes after ischemic stroke. These findings strongly indicate that microglial NR4A1 regulates TNF expression and stroke pathogenesis.

The work by Liu and colleagues is novel and intriguing as it identifies a previously unidentified role of NR4A1 as an RNA-binding protein, highlights a new regulatory mechanism of TNF expression in microglia, and underscores a key function of NR4A1 in microglial polarization and stroke outcomes. These findings provide new molecular targets with therapeutic potential. For example, NR4A1 may be up-regulated to decrease the production of pro-inflammatory cytokines in microglia and attenuate their pro-inflammatory polarization. This will likely ameliorate neuroinflammation and improve neurological symptoms in stroke and neurodegenerative disorders. Since pro-inflammatory microglia play an important role in pathogen clearance, NR4A1 up-regulation may aggravate injury in infectious diseases. In addition, the timing of NR4A1 activation may also affect disease progression and outcomes. Therefore, it is important to investigate the therapeutic effects of NR4A1 manipulation in various neurological diseases and at multiple time points.

Next, whether NR4A1 uses a similar mechanism to regulate the expression of other pro-inflammatory cytokines needs to be validated in vivo. Although both in vitro and in vivo studies support the hypothesis that NR4A1 negatively regulates TNF expression in microglia, evidence supporting NR4A1’s inhibitory role in the expression of other pro-inflammatory cytokines (e.g., IL-1β and IL-6) is only found in vitro. How to reconcile the different results of IL-1β and IL-6 expression in vitro and in vivo? Based on the observation that up-regulated NR4A1 is mainly found in microglial cytoplasm in vivo but in both cytoplasm and nucleus in vitro, the authors speculate that different pro-inflammatory cytokines are regulated by NR4A1 through distinct mechanisms. Specifically, cytoplasmic NR4A1 regulates TNF expression posttranscriptionally via binding to and destabilizing *TNF* mRNA, while nuclear NR4A1 regulates the expression of IL-1β and IL-6 at the transcriptional level as a transcription factor. This hypothesis is further confirmed by expressing NR4A1 exclusively in the cytoplasm. What confers the different subcellular distribution of NR4A1 in vitro and in vivo, however, remains unknown and should be explored in the future. This information will allow fine-tuning of neuroinflammation, which will likely enhance the efficacy and reduce the side effects of potential therapies.

In addition, it remains unclear which domain of NR4A1 binds to m^6^A sites and how exactly it interacts with m^6^A. Identifying the specific domains that bind to m^6^A-modified mRNAs and understanding the dynamics of such interaction will provide insights into the molecular mechanism of microglial activation and promote the development of novel treatments for various neurological diseases.

Finally, the translational potential of this study should be validated using human microglial cells in the future. Many studies report species-specific features of microglia [10,11]. Therefore, findings in rodents need to be validated in human cells. Although this study [9] confirmed up-regulation of NR4A1 in microglia after stroke in postmortem human brains, it is unclear whether NR4A1 modulates TNF expression via the same posttranscriptional mechanism in human microglia. Validating this observation in human microglia or microglia derived from induced pluripotent stem cells would significantly increase the translational potential of this work.
